# Supplementary material for: Coral restoration alters reef soundscapes but machine learning and manual analyses suggest different recovery rates
Source: PLoS One. 2026 May 27;21(5):e0347307. doi: 10.1371/journal.pone.0347307 (PMC13215545; doi:10.1371/journal.pone.0347307)
Supplement: Attachment — Submitted filename: Comments.docx [file pone.0347307.s002.docx]

**Coral restoration alters reef soundscapes but machine learning and manual analyses**

**suggest different recovery rates**

**Abstract:**

**Comments 1:** The abstract may be slightly lengthy for typical journal standards. Consider condensing it to emphasize the principal findings. Certain methodological details might be more appropriately relocated to the main body of the manuscript.

**Comment 2: Conceptual Link Between Reef Function, Health, and Soundscape Recovery**

### The manuscript would benefit from a clearer articulation of the conceptual relationship between reef function, ecological health, and soundscape recovery. The assumption that acoustic similarity reflects ecological recovery is currently implied but underdeveloped. A brief conceptual framework—either as a figure or succinct narrative—clarifying how elements of reef health (e.g., biodiversity, trophic dynamics) relate to acoustic signatures would strengthen the interpretation of PAM as a proxy for ecosystem function and contextualise the differing results from manual and machine learning approaches.

### ****Comment 3: Clarification of Hypotheses****

The hypotheses would benefit from greater specificity. The authors should clearly state their a priori expectations for each method—for example, whether manual analysis was expected to show similarity between restored and healthy sites, and whether machine learning would cluster the restored site with degraded or healthy reefs. This would strengthen the rationale for the dual-method approach and aid result interpretation.

**Methods**

**Comment 4:** The use of only one restoration site should be acknowledged as a limitation, as it restricts the generalisability of the findings. Including this in the discussion would provide appropriate context for the interpretation of results.

**Comment 5:** It would be helpful to clarify whether the designated “healthy” site was matched for key abiotic factors such as water flow, exposure, and depth, in order to minimise potential environmental confounding in soundscape comparisons.

**Comment 6:** The authors should provide a brief justification for the selection of the 1:4 duty cycle and the specific gain setting used during acoustic recordings. If based on pilot data or prior studies, a citation or explanation would be appropriate.

**Comment 7:** The rationale for limiting acoustic recordings to one hour per time phase should be explained. If this decision was influenced by logistical constraints (e.g., memory capacity or battery life), this should be stated.

**Comment 8:** Manual analysis was conducted by a single observer. The authors are encouraged to discuss the potential for observer bias and, if applicable, report intra-observer reliability or consistency.

**Comment 9:** Clarification is needed on whether any training materials, reference sound libraries, or prior validated datasets were used to guide the classification of the 15 fish call types. This would improve transparency and reproducibility.

**Comment 10:** While the use of UMAP is appropriate for visualising high-dimensional data, its outputs are difficult to interpret biologically. The authors should consider discussing the limitations of this approach and suggest the use of interpretable features in future work.

**Comment 11:** The decision to subsample 10 out of every 12 recordings should be justified, and any implications for sampling bias or representativeness should be acknowledged.

### ****Results****

**Comment 12: (Section 4.1 – Manual Detection):** The statistical methods are appropriate and clearly reported. However, the authors should place more emphasis on effect sizes to convey ecological relevance. Additionally, the exclusion of lunar phase from the richness model should be briefly justified.

**Comment 13: (Section 4.2 – Machine Learning):** The UMAP results are well visualised, but further interpretation is needed. The clustering of Full Moon recordings from the Healthy site with Degraded/Outplant sites should be explored—possibly linked to invertebrate activity. The authors should also state whether any acoustic indices (e.g., ACI, ADI) were considered to support or contrast the UMAP findings.

### ****Discussion****

**Comment 14: (Section 5 – Discussion):** The discussion is balanced and acknowledges the limitations of machine learning methods. However, the term “restoration-specific soundscape” should be more clearly defined in functional terms. The authors should also briefly discuss how this approach could be applied in conservation practice and consider comparing their findings with recent PAM-based studies (e.g., Jarriel et al. 2024; Azofeifa-Solano et al. 2025).

### ****References****

**Comment 15: (Section 7 – References):**

- The reference list is comprehensive and includes key literature in PAM, coral restoration, and machine learning in ecoacoustics.
- Inclusion of recent studies up to 2025 is commendable.
- Formatting should be reviewed for consistency, particularly in spacing and punctuation.
- Preprint citations (e.g., bioRxiv) should be checked for updated peer-reviewed versions and replaced where applicable.

**Comment 16: (Figures 1–5):**

- **Figure 1:** The site map is clear and well-labelled. However, the addition of a compass orientation and basic bathymetric cues would enhance spatial context and interpretability.
- **Figure 2:** The schematic effectively conveys the analytical workflow. To improve visual clarity, the authors should consider standardising formatting elements such as font size, spacing, and alignment.
- **Figure 3:** This figure presents the data clearly and supports the statistical findings well. Including effect sizes or confidence intervals would strengthen its interpretive value.
- **Figure 4:** The UMAP visualisation is well-constructed and demonstrates clustering effectively. The addition of ellipses or convex hulls to delineate groupings would improve interpretability.
- **Figure 5:** The clustering by lunar phase offers valuable insight. Enhancing legend formatting and providing clear subtitles for each panel would aid reader comprehension.
